# Whole‐genome sequencing identifies novel candidate pathogenic variants associated with left ventricular non‐compaction in a three‐generation family

**DOI:** 10.1002/ctm2.501

**Published:** 2021-08-09

**Authors:** Zhe Zhang, Shiying Li, Kun Wang, Zicheng Zhao, Heng Zhang, Shuaicheng Li, Xiaofei Jiang

**Affiliations:** ^1^ Department of Cardiology Zhuhai People's Hospital (Zhuhai Hospital Affiliated with Jinan University) Zhuhai Guangdong P. R. China; ^2^ Shenzhen Byoryn Technology Co., Ltd Shenzhen Guangdong P. R. China; ^3^ Department of Ultrasonography Zhuhai People's Hospital (Zhuhai Hospital Affiliated with Jinan University) Zhuhai Guangdong P. R. China; ^4^ Shenzhen Research Institute City University of Hong Kong Shenzhen Guangdong P. R. China

**Abbreviations**
CADD: combined annotation dependent depletion
FGF: fibroblast growth factor
LVNC: left ventricular non‐compaction
PCP: planar cell polarity
RA: retinoic acid
SNV: single‐nucleotide variant
WGS: whole‐genome sequencing

Dear Editor,

In this work, we found three novel candidate variants, namely a stop‐gain *ZNF107* (c.G1021T) and two missense variants *CYP26B1* (c.C364A) and *KIF16B* (c.G1748A), to be the most plausible causes for the left ventricular non‐compaction (LVNC) in a three‐generation Chinese family.

LVNC is a rare genetic cardiomyopathy with two morphological features: a thick bilayered myocardium with prominent ventricular trabeculations and deep intertrabecular recesses in the left ventricular wall.^1^ Reports on LVNC‐related genes are relatively limited, and approximately 42% of LVNC cases are familial, suggesting the existence of unknown mechanisms affecting the explored genetic etiologies of LVNC.^2^ Fibroblast growth factor (FGF) signaling is likely a contributing factor of LVNC for its pivotal role in compact myocardium proliferation.^3^ Experiments on mouse embryos showed that some FGF proteins have high expression levels in both the endocardium and epicardium, and their deficiencies lead to a thin myocardium wall and therefore non‐compaction.^4^ Moreover, excessive retinoic acid (RA), a metabolite of vitamin A, can interrupt the activation of the planar cell polarity (PCP) pathway regulating cardiomyocyte polarization.^5^ As a result, un‐polarized cardiomyocytes maintain the original round shape, leading to a non‐compacted myocardium. Several mouse models with deficiency in key PCP signaling components develop VNC.^6^

In this study, we recruited a three‐generation Chinese family suffering from LVNC following autosomal dominant inheritance (Figure 1A). The proband (WZYF13), a 47‐year‐old male, was hospitalized for complaining fatigue with edema of low limbs. Investigation into prior cardiac history yielded no indications for systemic diseases. However, echocardiography in Figure 1B revealed a 65‐mm end‐diastolic diameter of the left ventricle with the global ejection factor as 28%. We observed a thin, compacted layer, increased and eminent trabeculation in the left ventricle, and deep intertrabecular recesses with blood perfusion. Doppler echocardiography presented mild regurgitation of mitral and aortic valves but showed no other valve abnormalities. Based on the above clinical results, the proband was diagnosed with LVNC. Subsequently, relatives of the proband undertook a comprehensive clinical history review, physical examination, and echocardiography. The proband's monozygotic twin (WZYF13, Figure 1C) and 6‐year‐old daughter (WLQC12‐gb1, Figure 1D) also met the echocardiographic LVNC diagnostic criteria. Other examined family members showed no signs of LVNC (Table S1).

**FIGURE 1:**
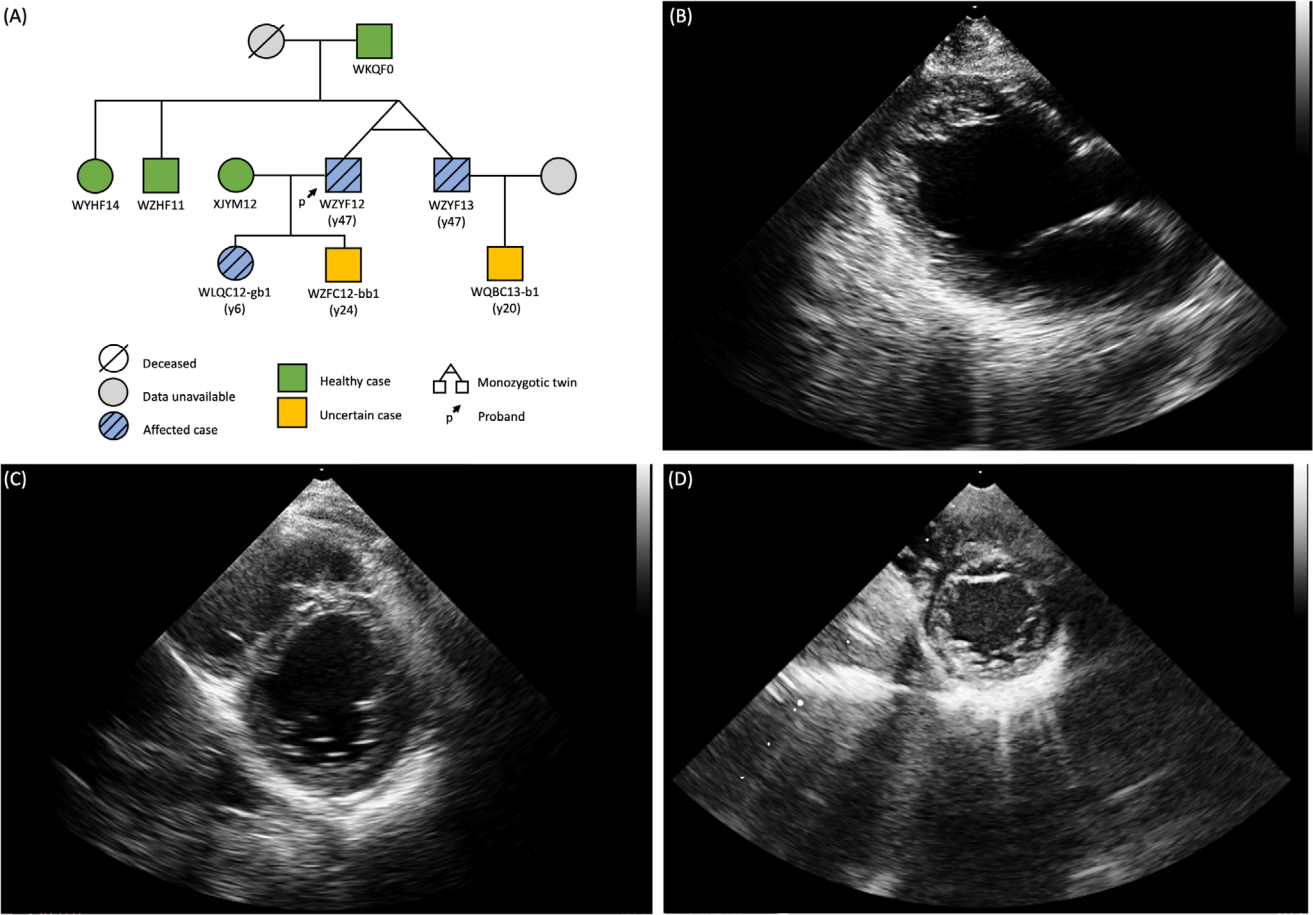
The family pedigree and the echocardiographic images of the twins and the girl suffering from LVNC. (A) Pedigree of the 3‐generation family. Healthy, uncertain, and affected cases are colored green, orange, and blue, respectively. An arrow marks the proband WZYF12. WZYF12 and WZYF13 are monozygotic twins. (B) Echocardiography of WZYF12 with ratio between non‐compacted and compacted myocardium in telediastole larger than 2 in the apex. (C) Echocardiography of WZYF13 showing a global EF of 43%, moderate mitral, and mild tricuspid valves regurgitation. Unlike previous echocardiography, it also presented non‐compacted myocardium in the apex and lateral wall of the left ventricle. Upon further examination, the twin of the proband also matched the echocardiographic diagnostic criteria for LVNC. (D) Echocardiography of WLQC12‐gb1 meeting the echocardiographic LVNC diagnostic criteria. Abbreviations: EF, ejection fraction; LVNC, left ventricular non‐compaction

We performed whole‐genome sequencing (WGS) on all available family members detailed in the Supplementary Methods. We identified no copy number events in autosomal chromosomes segregating with the affected cases and healthy cases (Figure S1). Moreover, 37 single‐nucleotide variants (SNVs), consisting of 34 exonic variants and three splicing variants (Table S2), remained after the filtering process in Figure 2. The three splicing variants are a stop‐gain in *ZNF107*, a frameshift deletion in *ARHGAP45*, and a non‐frameshift deletion in *PRR22*, with scaled Combined Annotation Dependent Depletion (CADD)^7^ scores of 31, 6.646, and 5.532, respectively. While the *ARHGAP45* and *PRR22* variants fail to meet the suggested scaled CADD score threshold for deleterious variants, the *ZNF10* variant is classified as the top 0.1% deleterious variants of the human genome. *ZNF107* encodes a protein with several C2H2‐type zinc finger regions that serve as transcriptional regulators mediating direct DNA interactions. Although the physiological function of *ZNF107* remains largely unexplored, it shared similar C2H2‐type zinc‐finger regions with several proteins related to cardiac functions and congenital heart diseases^8^ and thus has potential implications in cardiac functions.

**FIGURE 2:**
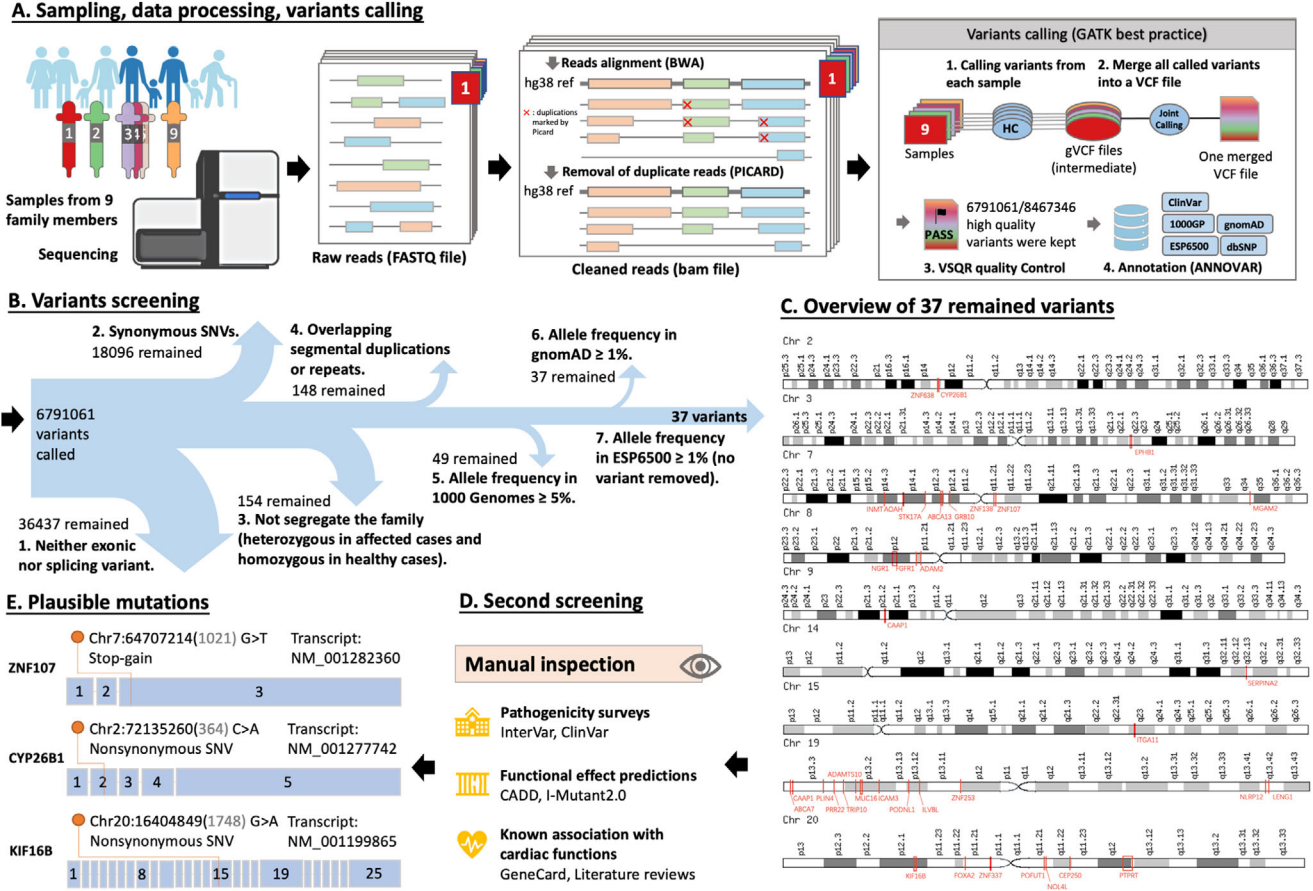
Overview of our method. (A) We obtained raw reads files from sequenced samples of nine family members. We aligned raw reads with BWA and removed duplicate reads marked by PICARD. We applied GATK's joint calling module on cleaned reads files, resulting in a single VCF file of 8467346 merged variants. In GATK's VSQR module, high‐quality variants are flagged as “PASS”, and other variants are filtered. We annotate the high‐quality variants by ANNOVAR. (B) We filtered variants with an array of constraints. (C) The overview of 37 possibly deleterious variants on chromosomes. (D) Manual inspections of the 37 variants. **(E)** We identified three possibly plausible variants

Among exonic variants, fourteen were classified as likely benign by the ACMG/AMP 2015 guideline and ClinVar. Furthermore, one variant is found in only one of four datasets and thus is unreliable. Consequently, we exclude the above 15 variants from further analysis. Of the other nineteen possible disease‐causing variants, 11 have scaled CADD scores between 20 and 30, indicating the variants are classified as the top 1%–0.1% deleterious variants of the human genome. Furthermore, nine of the 11 variants have ΔΔG < −0.5 which indicate a decrease in protein stability (Table S3). In particular, three variants on CYP26B1, PODNL1, and KIF16B are predicted to induce large stability decrease with ΔΔG values of −1.45, −1.42, and −1.37, respectively. While PODNL1 has limited associations with cardiac functions, the other two genes both play significant roles in pathways implicated in myocardium compaction. KIF16B encodes a kinesin‐like protein mediating FGF signal transduction via regulating the surface presentation of FGFR2. Ueno et al showed that the developmental phenotypes of KIF16B‐deficient mouse embryos and embryoid bodies imitate FGFR2 deficiency, which results in a thin myocardium.^6^ *CYP26B1* encodes a member of the CYP26 family (Cyp26a1, Cyp26b1, Cyp26c1) which is the primary mechanism to limit the tissue concentration of RA. Zebrafish embryo models with Cyp26a1 and Cyp26c1 deficiency developed more un‐polarized and separated ventricular cardiomyocytes.^9^ High expression level of Cyp26b1 is related to atherosclerotic lesions,^10^ while not yet related to cardiac functions.

The variant on *CYP26B1* (c.C364A) is highly conserved (CADD conservation = 1.0 from 140 aligned protein sequences) and is predicted as deleterious by SIFT, Polyphen2 (HDIV/HVAR), LRT, MutationTaster, MutationAssessor, MetaSVM/LR, and M‐CAP. The variant induces a mutation at Leucine 122 (L122) in CYP26B1 to Methionine (L122M). While Methionine is not yet related to muscle functions, Leucine is highly related to muscle health since it promotes protein synthesis, decreases protein breakdown during physical trauma, and accelerates muscle recovery. The variant also introduced a new hydrogen bond between L122M and 179K (Figure 3), where the addition of hydrogen bonds has a potential effect on protein folding. Moreover, the hydrogen bond adds a new binding force between two *α*‐helixes, possibly having a potent effect on protein folding.

**FIGURE 3:**
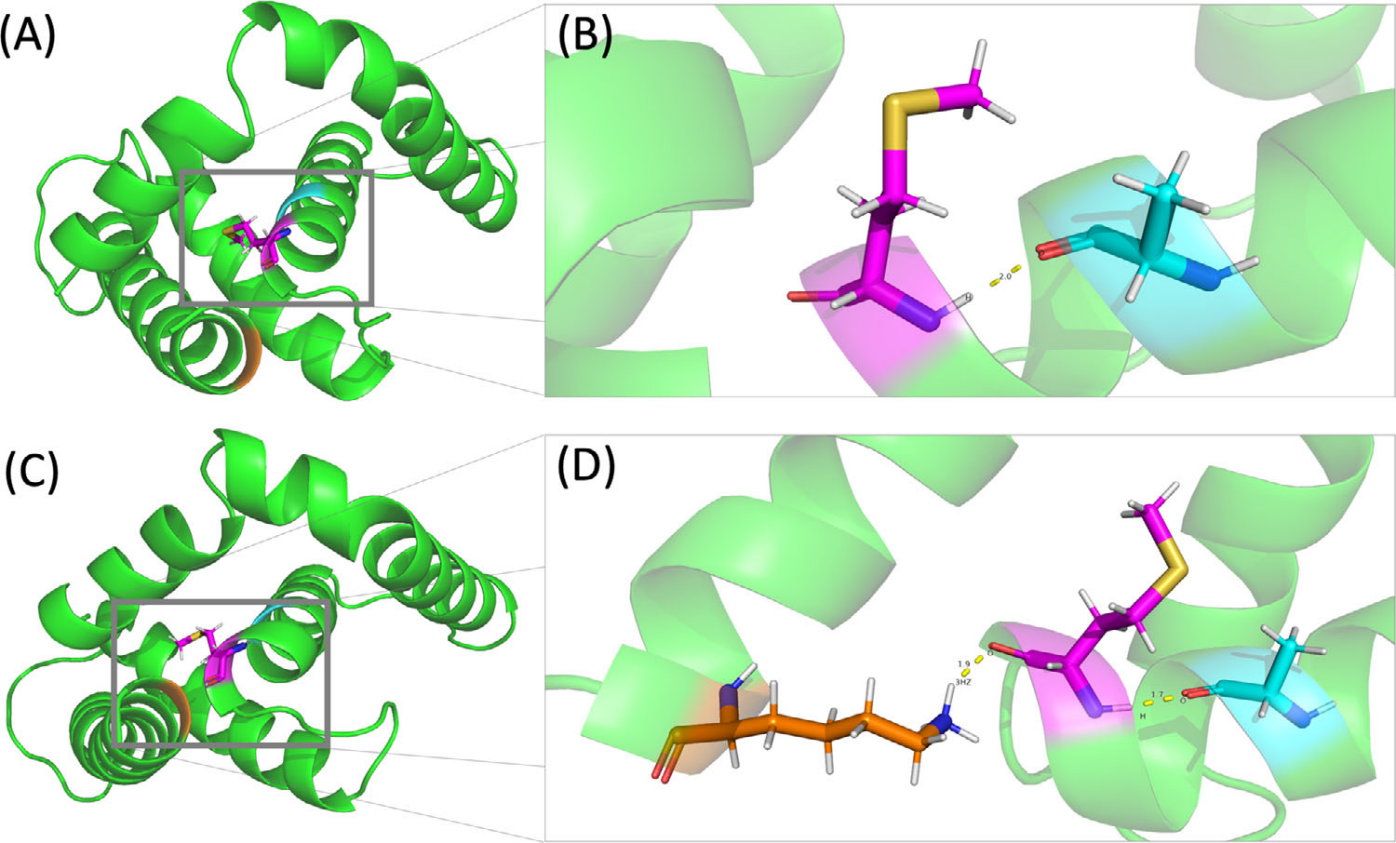
Mutation site and structure of CYP26B1's partial amino acid sequence (61‐182) visualized by PyMol, showing structural difference between wide type and mutant. (A) Wild type of CYP26B1 (61‐182) predicted by Robetta, highlighting the site of 122L at the end of an *α*‐helix. (B) A closer view of site 122L. A hydrogen bond (yellow dotted lines) is formed between 122L and 118A with a distance of 2.0Å. (C) Mutant of CYP26B1 (61–182) predicted by Robetta introducing a single amino acid residue replacement L122M. A distortion of at the end of *α*‐helix 3 toward the end of *α*5 can be observed. (D) A closer view of mutation site M122L. Another hydrogen bond is formed between L122M of α3 and 179K of *α*5 with a distance of 1.9Å

In conclusion, our results suggested that the three novel SNVs revealed here may be associated with LVNC; however, further study is required to consolidate the results. In clinical practice, WGS is becoming more accessible, efficient, and effective. For genetic diseases like LVNC, prompt heritage analysis on the whole family is valuable for early diagnosis and treatment. In this case, the 6‐year‐old daughter of the proband with LVNC will receive regular following‐up to monitor any possible symptoms.

## CONFILICT OF INTEREST

The authors declare that they have no conflict of interest.

## ETHICS STATEMENT

The study was approved by the Ethics Committee of Zhuhai People's Hospital.

## AUTHOR CONTRIBUTIONS

Xiaofei Jiang coordinated the study. Zhe Zhang conceived the study. Zhe Zhang and Xiaofei Jiang performed all medical examinations on family members. Heng Zhang provided insights from the ultrasound aspects. Zhe Zhang and Kun Wang collected the samples. Zhe Zhang and Zicheng Zhao concerted genome sequencing data acquisition and delivery. Shuaicheng Li advised the data analyses and interpretation. Shiying Li performed the data analyses and computer program writing. Zhe Zhang, Shiying Li, Shuaicheng Li, and Xiaofei Jiang wrote the manuscript and formatted all figures. All the authors have proofread the manuscripts.

## DATA AVAILABILITY STATEMENT

The variation data reported here have been deposited in the Genome Variation Map (GVM) in Big Data Center, Beijing Institute of Genomics (BIG), Chinese Academy of Science, with accession number GVM000116 at http://bigd.big.ac.cn/gvm/getProjectDetail?project=GVM000116.

## Supporting information



Figure S1. Copy number variation identified in nine family members.

Table S1. Table of clinical evaluation on nine family members.

Table S2. Table of 37 variants remained after filtering.

Table S3. Table of nine possible disease‐causing variants.

Supplementary Methods. The detailed materials and methods in the study.
