# Supplementary figures and images for: Whole‐genome sequencing identifies novel candidate pathogenic variants associated with left ventricular non‐compaction in a three‐generation family
Source: Clin Transl Med. 2021 Aug 9;11(8):e501. doi: 10.1002/ctm2.501 (PMC8351521; doi:10.1002/ctm2.501)

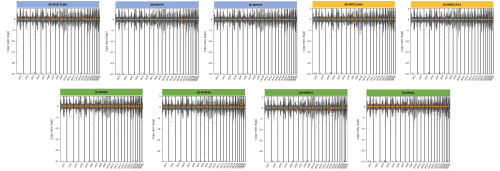

Supplement: Supplementary file 1 — Figure S1. Copy number variation identified in nine family members. [file CTM2-11-e501-s005.TIF]
