# Supplementary material for: Whole‐genome sequencing identifies novel candidate pathogenic variants associated with left ventricular non‐compaction in a three‐generation family
Source: Clin Transl Med. 2021 Aug 9;11(8):e501. doi: 10.1002/ctm2.501 (PMC8351521; doi:10.1002/ctm2.501)
Supplement: Supplementary file 2 — Table S1. Table of clinical evaluation on nine family members. [file CTM2-11-e501-s003.pdf]

TABLE S1 Table of clinical evaluation on nine family members.

Generation	Individuals	Sex	Age	Clinical history	Echocardiography NC/C*	IVSD	LVEDD	EF(%)	LVNC Present	Variants *ZNF107* (c.G1021T)	*CYP26B1* (c.C364A)	*KIF16B* (c.G1748A)
I	WKQF0	M	80	healthy	-	7	48	64	none	0/0	0/0	0/0
II	WYHF14	F	51	healthy	-	11	45	70	none	0/0	0/0	0/0
II	WZHF11	M	50	hypertension, obesity	-	13	60	53	none	0/0	0/0	0/0
II	WZYF12	M	47	heart failure with edeme of lower limbs	2.2	10	65	28	affected and meets diagnostic criteria for LVNC	0/1	0/1	0/1
II	XJYM12	F	46	none	-	7	40	67	none	0/0	0/0	0/0
III	WLQC12-gb1	F	6	closure of atrial spetal defect at 4-year old	2.1	7	29	70	none but meets diagnostic criteria for LVNC	0/1	0/1	0/1
III	WZFC12-bb1	M	21	healthy	-	7	54	55	none	0/0	0/1	0/0
II	WZYF13	M	47	alcohol abuse history and quitted 7 years ago; suffered heart failure 7 years ago and followed the treatment plan	2.3	10	69	43	affected and meets diagnostic criteria for LVNC	0/1	0/1	0/1
III	WQBC13-b1	M	22	obesity	-	11	44	48	none	0/0	0/1	0/1

NC/C, non-compaction to compaction ratio; IVSD, interventricular septum at end diastole; LVEDD, left ventricular internal dimensions at end diastole; EF, indicates left ventricular ejection fraction; LVNC, left ventricular non-compaction.
