# Supplementary material for: Whole‐genome sequencing identifies novel candidate pathogenic variants associated with left ventricular non‐compaction in a three‐generation family
Source: Clin Transl Med. 2021 Aug 9;11(8):e501. doi: 10.1002/ctm2.501 (PMC8351521; doi:10.1002/ctm2.501)
Supplement: Supplementary file 3 — Table S2. Table of 37 variants remained after filtering. [file CTM2-11-e501-s001.pdf]

TABLE S2 Table of 37 variants remained after filtering.

							Pathogenecity Predictions			Allele Frequency					
Gene	Chr	Pos	Ref	Alt	Type	rs-code	Intervar	ClinVar	CADD	ExAC	ExAC(EAS)	1000GP	ESP6500	gnomAD	gnomAD(eas)
Splicing Variants (3)															
ZNF107	chr7	64707214	G	T	stopgain	rs184622647	Uncertain significance	.	31	0.0007	0.0094	0.00159744	.	0.0002	0.0083
ARHGAP45	chr19	1077769	G	-	frameshift deletion	.	.	.	6.646	.	.	.	.	0.00002799	0.0013
PRR22	chr19	5783296	CTG	-	nonframeshift deletion	rs763758443	.	.	5.532	0.00000854	0	.	0.0029	.	.
Possibly Deterious SNPs (11)															
EPHB1	chr3	135249358	C	T	nonsynonymous SNV	rs143309901	Uncertain significance	.	28.3	0.0003	0.0037	0.000599042	.	0.0000907	0.0032
TRIP10	chr19	6746156	C	T	nonsynonymous SNV	rs759521484	Uncertain significance	.	27.6	0.0003	0	.	.	0.00001397	0.0003
CYP26B1	chr2	72135260	G	T	nonsynonymous SNV	rs779832742	Uncertain significance	.	26.2	0.0001	0.0015	.	.	0.00002791	0.0013
PODNL1	chr19	13933228	A	C	nonsynonymous SNV	rs193206432	Uncertain significance	.	26.2	0.0003	0.0144	0.000399361	.	0.0001	0.0057
PTPRT	chr20	42352152	G	T	nonsynonymous SNV	rs552058956	Uncertain significance	.	26.1	0.0003	0.0036	0.000599042	.	0.0001	0.0057
ILVBL	chr19	15115592	T	G	nonsynonymous SNV	rs760981000	Uncertain significance	.	24.1	0.00004956	0.0007	.	.	0.00004187	0.0019
FOXA2	chr20	22582968	C	T	nonsynonymous SNV	rs199654135	Uncertain significance	.	23.7	0.0003	0.0036	0.000599042	.	0.00008376	0.0029
STK17A	chr7	43623824	G	C	nonsynonymous SNV	rs3779062	Uncertain significance	.	23.3	0.0032	0.0424	0.00798722	.	0.0008	0.0313
NLRP12	chr19	53811043	G	A	nonsynonymous SNV	rs111754022	Uncertain significance/ Likely pathogenic	Conflicting interpretations of pathogenicity	23.3	0.00006604	0	.	.	0.00002094	0
KIF16B	chr20	16404849	C	T	nonsynonymous SNV	rs144884167	Uncertain significance	.	23.3	0.0052	0.0254	0.00958466	0.0012	0.0027	0.0243
ITGA11	chr15	68328237	C	T	nonsynonymous SNV	.	Uncertain significance	.	21.8	.	.	.	.	.	.
Borderline SNPs (8)															
ICAM3	chr19	10334346	G	A	nonsynonymous SNV	.	Uncertain significance	.	16.87	.	.	.	.	.	.
AOAH	chr7	36632061	C	T	nonsynonymous SNV	rs3735384	Uncertain significance	.	15.84	0.0056	0.0402	0.00858626	0.0002	0.0031	0.0428
ABCA7	chr19	1046401	C	G	nonsynonymous SNV	rs201932817	Uncertain significance	.	13.23	0.0002	0.0025	0.00139776	.	0.00008371	0.0029
ZNF638	chr2	71422836	A	G	nonsynonymous SNV	rs61739715	Uncertain significance	.	13.1	0.0068	0.0589	0.00898562	0.0004	0.0034	0.0533
LENG1	chr19	54155824	G	A	nonsynonymous SNV	rs375647598	Uncertain significance	.	5.788	0.0009	0.0001	0.000399361	0.0002	0.0002	0
PLIN4	chr19	4511054	G	A	nonsynonymous SNV	rs185740143	Uncertain significance	.	1.599	0.0005	0.0071	0.00299521	.	0.0002	0.0099
MUC16	chr19	8939315	G	A	nonsynonymous SNV	rs139208500	Uncertain significance	.	1.482	0.0021	0.0255	0.00658946	0.0002	0.001	0.0345
MGAM2	chr7	142221912	A	T	nonsynonymous SNV	rs186809625	Uncertain significance	.	1.351	.	.	0.000798722	.	0.0001	0.0057
Likely Benign SNPs (15)															
POFUT1	chr20	32230834	A	G	nonsynonymous SNV	rs79984831	Likely benign	Benign	19.93	0.0036	0.0469	0.00838658	.	0.0011	0.0434
NOL4L	chr20	32452323	C	T	nonsynonymous SNV	rs374479145	Likely benign	.	19.83	0.00004233	0	.	.	0.00001396	0
INMT	chr7	30752287	A	C	nonsynonymous SNV	rs77743549	Likely benign	.	19.44	0.0082	0.0199	0.00479233	0.0052	0.0062	0.0147
CAAP1	chr9	26892525	A	G	nonsynonymous SNV	rs3739549	Likely benign	.	19.03	0.001	0.0193	0.00299521	.	0.0003	0.0128
NRG1	chr8	31640208	T	C	nonsynonymous SNV	rs199941564	Likely benign	.	18.45	0	0	.	.	0.0005	0.0219
ABCA13	chr7	48272206	G	T	nonsynonymous SNV	rs117711958	Likely benign	.	17.65	0.0019	0.0256	0.00499201	0.000086	0.0007	0.0249
ZNF138	chr7	64831657	T	A	nonsynonymous SNV	rs551808152	Likely benign	.	16.78	0.0005	0.0065	0.000798722	.	0.0002	0.0067
ADAMTS10	chr19	8605091	C	T	nonsynonymous SNV	rs3814291	Likely benign	Benign/Likely benign	15.91	0.0093	0.0162	0.0111821	0.0006	0.0046	0.0115
FGFR1	chr8	38429720	G	A	nonsynonymous SNV	rs140382957	Likely benign	Benign/Likely benign	15.82	0.0025	0.0454	0.00399361	0.000077	0.0004	0.0176
ZNF337	chr20	25676476	C	T	nonsynonymous SNV	rs147339647	Likely benign	.	14.59	0.0008	0.0117	0.00219649	.	0.0004	0.0135
ZNF253	chr19	19891764	A	G	nonsynonymous SNV	rs77010331	Likely benign	Benign	13.21	0.0066	0.0711	0.0139776	0.0003	0.0021	0.0565
CEP250	chr20	35493524	A	T	nonsynonymous SNV	rs2296403	Likely benign	Benign	12.5	0.0035	0.0469	0.0101837	.	0.0012	0.0501
ADAM2	chr8	39838168	A	C	nonsynonymous SNV	rs199802183	Likely benign	.	0.1	0.0007	0.0096	0.00279553	.	0.0003	0.0121
GRB10	chr7	50674515	G	A	nonsynonymous SNV	rs80244589	Likely benign	.	0.002	0.0007	0.0085	0.000998403	.	0.0002	0.0089
SERPINA2	chr14	94364435	T	C	unknown	.	.	.	10.38	.	.	.	.	0.000006978	0.0003
