# Supplementary material for: Whole‐genome sequencing identifies novel candidate pathogenic variants associated with left ventricular non‐compaction in a three‐generation family
Source: Clin Transl Med. 2021 Aug 9;11(8):e501. doi: 10.1002/ctm2.501 (PMC8351521; doi:10.1002/ctm2.501)
Supplement: Supplementary file 4 — Table S3. Table of nine possible disease‐causing variants. [file CTM2-11-e501-s002.pdf]

TABLE S3 Table of nine possible disease-causing variants.

Gene	Chr	Pos	Ref	Alt	CADD	I-Mutant2.0	Functional Effect Detailed Results										
							SIFT	Polyphen2 (HDIV)	Polyphen2 (HVAR)	LRT	Mutation Taster	Mutation Assessor	FATHMM	PROVEAN	Meta SVM	Meta LR	M-CAP
CYP26B1	chr2	72135260	G	T	26.2	-1.45	D	D	D	D	D	M	T	N	D	D	D
PODNL1	chr19	13933228	A	C	26.2	-1.42	D	D	D	U	D	H	D	D	D	D	.
KIF16B	chr20	16404849	C	T	23.3	-1.37	T	P	B	D	D	N	T	N	T	T	.
PTPRT	chr20	42352152	G	T	26.1	-0.91	D	P	B	D	D	M	T	N	T	T	D
STK17A	chr7	43623824	G	C	23.3	-0.78	T	P	P	N	D	N	T	N	T	T	.
TRIP10	chr19	6746156	C	T	27.6	-0.69	D	D	D	U	D	M	T	D	T	T	D
NLRP12	chr19	53811043	G	A	23.3	-0.66	T	D	D	N	N	M	T	N	T	T	T
ITGA11	chr15	68328237	C	T	21.8	-0.66	T	B	B	D	D	L	T	N	T	T	T
EPHB1	chr3	135249358	C	T	28.3	-0.57	D	D	P	D	D	M	T	D	T	T	D

Variants are ordered descendently by I-Mutant2.0 scores. We colored pathogenic, likely pathogenic, benign, and unknown prediction results in the Functional Effect Detailed Results columns, red, pink, green, and yellow.
