# Supplementary material for: Whole‐genome sequencing identifies novel candidate pathogenic variants associated with left ventricular non‐compaction in a three‐generation family
Source: Clin Transl Med. 2021 Aug 9;11(8):e501. doi: 10.1002/ctm2.501 (PMC8351521; doi:10.1002/ctm2.501)
Supplement: Supplementary file 5 — Supplementary Methods. The detailed materials and methods in the study. [file CTM2-11-e501-s004.pdf]

SUPPLEMENTARY METHODS

Sample Collection and DNA Sequencing

5 ml whole blood was collected by the anticoagulant tube with EDTA. All samples was stored at -80°C for DNA extraction. Sequencing was conducted at Novogene Co. Ltd. on an Illumina HiSeq 2000 platform with 30x sequencing depths, 150 bp paired-end reads.

Clinical Evaluation

All nine available family members are interviewed for family history and medical records and received genetic testing and clinical assessments, including physical exam, electrocardiography, and echocardiography. The diagnosis of LVNC followed the echocardiographic diagnostic criteria: (1) a ratio of non-compacted to compacted (NC/C) subepicardial layer at end systole for a two-layered endocardium > 2 in the apex; (2) deep endomyocardial trabeculations in the left ventricle; and (3) deep recesses filled with blood as visualized on color Doppler imaging[1]. Besides, the left ventricular systolic dysfunction with an ejection fraction (EF) is < 30%. While not yet diagnosed for LVNC, individual WZFC12-bb1 and WQBC13-b1 are young (aged 24 and 20 respective) and might develop LVNC in the future. On the other hand, all unaffected individuals in the first and the second generation are around 50 years old and older, and should have developed LVNC symptoms if affected. Therefore, WZFC12-bb1 and WQBC13-b1 are classified as uncertain while other unaffected individuals as healthy. Analysis of the three-generation family's pedigree in **Fig. 1A** revealed the possibility of an autosomal dominant inheritance pattern of LVNC. Echocardiography of the affected twins and 6-year old girl are shown in **Fig. 1A**(B-D). **Supplementary Table 1** summarizes the detailed results for clinical evaluation.

Genetic Study

Sequenced reads were mapped to the human reference genome assembly GRCh38 using the Burrows-Wheeler-Alignment (BWA) tool. Duplicate reads were removed by PICARD. Small variants (SNPs and short indels) are called respectively from samples and merged by GATK4. The VQSR module of GATK4 facilitated quality control of the called variants. In total, 6,791,061 high-quality variants were called from all sequenced samples.

Among all called variants, we consider exonic or splicing SNPs that are either nonsense or missense for further analysis as they are most likely to affect gene functions in all types of SNPs. Additionally, given that LVNC is a relatively rare autosomal dominant disease, the possibility of both parents being affected is negligibly low. Therefore, the disease-causing variants should be heterozygous in affected cases and homozygous in healthy cases. Lastly, with the increasing prevalence estimates, the disease-causing variant may have a higher allele frequency in the general population than estimated in the past published work.

Before filtering SNPs with the above assumptions, we first used ANNOVAR (Version 2019Jun17) to annotate identified variants. Allele frequencies of variants in whole-genome and whole-exome data come from the latest 1000 Genomes Project dataset (1000GP), Exome Aggregation Consortium dataset (ExAC), gnomAD database, and the dataset from the NHLBI-ESP project with 6500 exomes (ESP6500). Subsequently, we filtered variants by the following criteria based on the above assumptions: (1) exonic or splicing variant; (2) nonsense or missense variant; (3) heterozygous in affected cases and homozygous in healthy cases; (4) overlapping neither segmental duplications nor repeats; (5) allele frequency in 1000GP $\leq 5\%$ or not reported; (6) allele frequency in gnomAD $\leq 1\%$ or not reported; (7) allele frequency in ESP6500 $\leq 1\%$ or not reported. Variants remained after filtering are potentially plausible and require further manual inspections. The pathogenicity of variants are first evaluated under the guidelines of American College of Medical Genetics (ACMG) and Association for Molecular Pathology (AMP)[2] using the InterVar software[3], along with their clinical significance reported by ClinVar[4]. Variants' potential effects on protein structures

and stability changes were surveyed by the Combined Annotation-Dependent Depletion (CADD) version 1.6 scores[5] and the Free Energy change value ($\Delta\Delta G$) using I-Mutant2.0[6] respectively. The evidence of impaired cardiac functions comes from both known associations with the heart and the expression levels of RNA and protein in the heart from GeneCard.

We also performed copy-number analysis as CNVs have also been reported as clinically significant to LVNC. Ceyhan-Birso et al. identified a clinically significant whole gene duplication of *PKP2* in a patient with LVNC[7]. Scott et al. also found a causative single-gene deletion of *NONO* in an infant suffering from severe LVNC[8]. Consequently, we interrogate the called variants and WGS data for all available family members to find clinically relevant CNVs using the Germline Analysis module of CNVkit. We excluded copy number (CN) events in sex chromosomes as LVNC has an autosomal dominant heritage pattern in the family.

REFERENCES

1. Jenni R, Oechslin E, Schneider J, Attenhofer Jost C, Kaufmann PA. Echocardiographic and pathoanatomical characteristics of isolated left ventricular non-compaction: A step towards classification as a distinct cardiomyopathy. *Heart* 2001. doi: 10.1136/heart.86.6.666

2. Richards S, Aziz N, Bale S, et al. Standards and guidelines for the interpretation of sequence variants: a joint consensus recommendation of the American College of Medical Genetics and Genomics and the Association for Molecular Pathology. *Genetics in Medicine* 2015; 17(5): 405-423. doi: 10.1038/gim.2015.30

3. Li Q, Wang K. InterVar: Clinical Interpretation of Genetic Variants by the 2015 ACMG-AMP Guidelines. *American journal of human genetics* 2017; 100(2): 267-280. 28132688[pmid]doi: 10.1016/j.ajhg.2017.01.004

4. Landrum MJ, Lee JM, Benson M, et al. ClinVar: public archive of interpretations of clinically relevant variants. *Nucleic acids research* 2016; 44(D1): D862–D868.

5. Rentzsch P, Witten D, Cooper GM, Shendure J, Kircher M. CADD: predicting the deleteriousness of variants throughout the human genome. *Nucleic Acids Research* 2018; 47(D1): D886-D894. doi: 10.1093/nar/gky1016

6. Capriotti E, Fariselli P, Casadio R. I-Mutant2.0: predicting stability changes upon mutation from the protein sequence or structure. *Nucleic acids research* 2005; 33(Web Server issue): W306-W310. 15980478[pmid]doi: 10.1093/nar/gki375

7. Ceyhan-Birsoy O, Pugh TJ, Bowser MJ, et al. Next generation sequencing-based copy number analysis reveals low prevalence of deletions and duplications in 46 genes associated with genetic cardiomyopathies. *Molecular Genetics & Genomic Medicine* 2016; 4(2): 143–151. doi: 10.1002/mgg3.187

8. Scott DA, Hernandez-Garcia A, Azamian MS, et al. Congenital heart defects and left ventricular non-compaction in males with loss-of-function variants in NONO. *Journal of Medical Genetics* 2017; 54(1): 47–53. doi: 10.1136/jmedgenet-2016-104039
